# Thoracic degenerative spondylolisthesis-associated myelopathy: A case report

**DOI:** 10.1097/MD.0000000000026150

**Published:** 2021-05-28

**Authors:** Huafeng Wang, Fengfei Lin, Guiqing Liang, Boling Liu, Yuhan Lin

**Affiliations:** Department of Spine Surgery, Fuzhou Second Hospital Affiliated to Xiamen University, Fuzhou, China.

**Keywords:** case report, degenerative spondylolisthesis, surgical decompression, thoracic myelopathy, thoracic spine

## Abstract

**Rationale:**

The thoracic spine is stabilized in the anteroposterior direction by the rib cage and the facet joints, thus thoracic degenerative spondylolisthesis is very uncommon. Here, we report a rare case of thoracic degenerative spondylolisthesis in which the lower thoracic region was the only region involved.

**Patient concerns:**

We present the case of a 56-year-old Chinese female who suffered from thoracic degenerative spondylolisthesis. She had a 2-year history of gait disturbance and bilateral lower-extremity numbness. The initial imaging examinations revealed Grade I anterior spondylolisthesis and severe cord compression, as well as bilateral facet joint osteoarthritis at T11/12.

**Diagnosis:**

The patient was diagnosed with thoracic degenerative spondylolisthesis-associated myelopathy.

**Interventions:**

She underwent a posterior decompression with transforaminal thoracic interbody fusion (TTIF) at T11/12.

**Outcomes:**

The patient recovered well after the operation, and MRI at 12-month follow-up revealed that spinal cord compression was relieved and high signal intensity in T2-weighted image was improved.

**Lessons:**

To the best of our knowledge, this is the first reported case of thoracic degenerative spondylolisthesis in which the lower thoracic region was the only region involved. Disruption of joint capsule, instability with micromotion, and degenerative disc may contribute to this rare disease. Posterior decompression with posterolateral fusion or TTIF were the main treatment modalities, however, TTIF has its unique advantages because of sufficient decompression, immediate stability and high fusion rate.

## 1 Introduction

The thoracic spine is stabilized in the anteroposterior direction by the rib cage and the facet joints, thus thoracic degenerative spondylolisthesis is more than uncommon.^[1–3]^ To the best of our knowledge, there have been only 10 cases reported in the English literature. All the reported cases of thoracic spondylolisthesis have occurred in people of Asian ethnicity. However, most of them were concomitant with lumbar spondylosis or diffuse idiopathic skeletal hyperostosis (DISH).^[2,3]^ Here we report a case of thoracic degenerative spondylolisthesis-associated myelopathy in which the lower thoracic region was the only region involved and to discuss the mechanism of thoracic spine spondylolisthesis.

## 2 Case presentation

**Figure 1:**
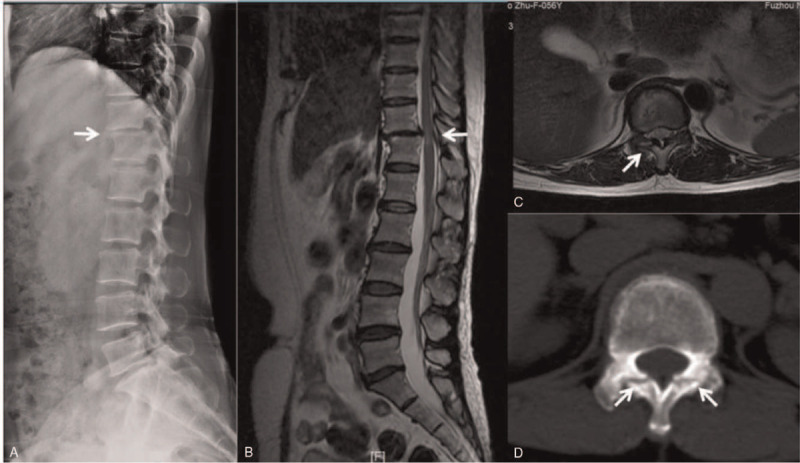
The imaging examinations at the time of presentation. A Sagittal X-ray (A) shows grade I anterior spondylolisthesis, a sagittal T2-weighted image (B) shows severe cord compression, an axial T2-weighted image (C) shows right facet joint hyperintense signal suggesting joint effusion, and an axial CT (D) show bilateral facet joint osteoarthritis at T11/12.

**Figure 2:**
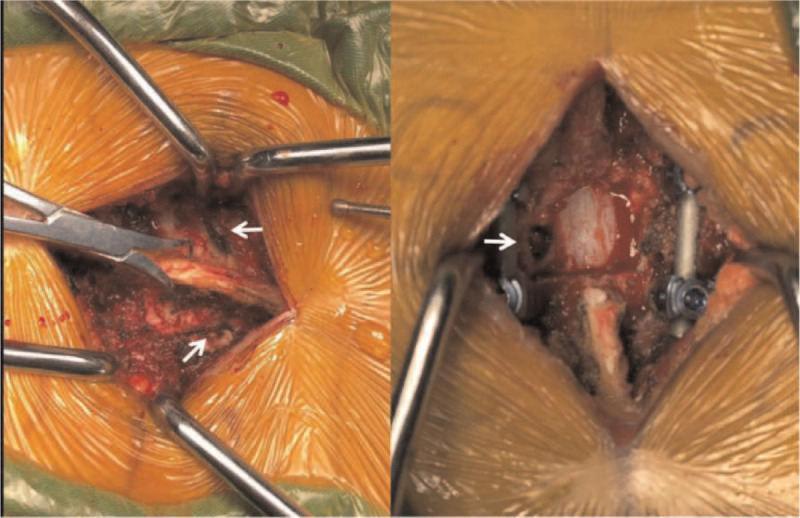
Intraoperative findings. Left: a gap in the facet joint, indicating disruption of the joint capsule. Right: the entire inferior articular process and superior portion of the superior articular process on the left side were removed.

**Figure 3:**
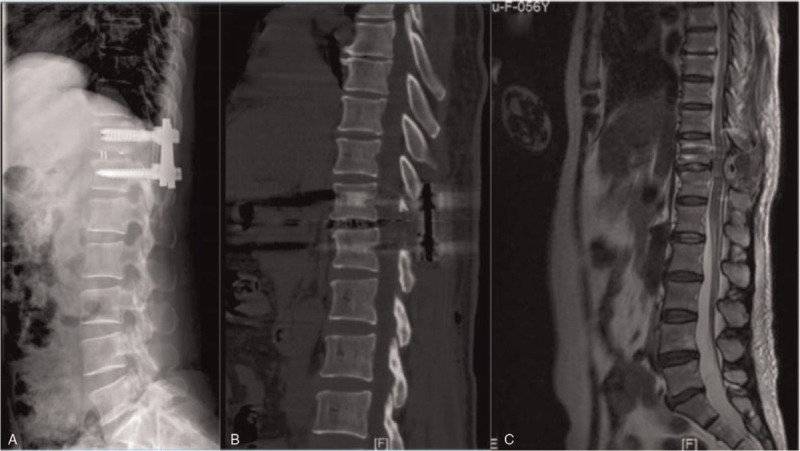
Postoperative images revealed that reduction of anterior displacement and resolution of the spinal cord compression.

**Figure 4:**
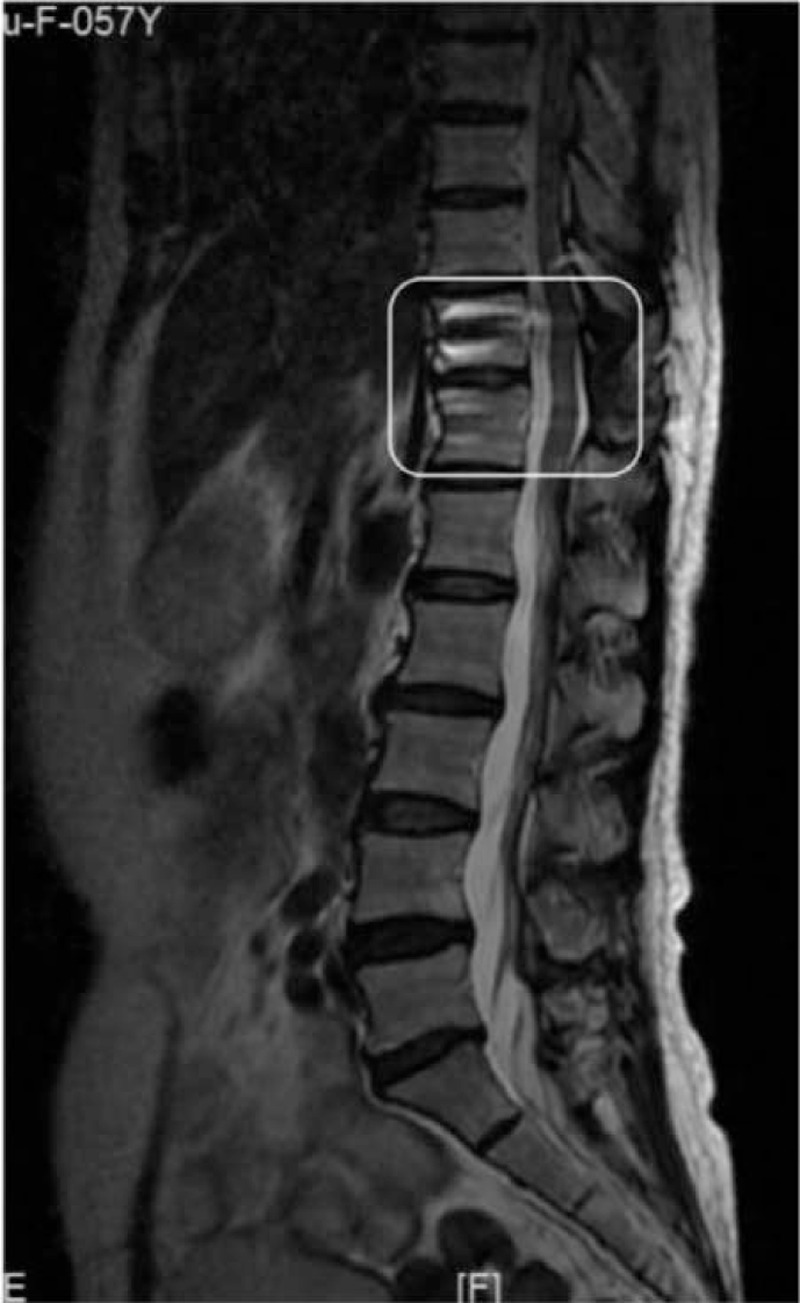
A sagittal T2-weighted image at 12-month follow-up shows an improvement in the high signal intensity on the T2-weighted image.

A 56-year-old Chinese woman visited our hospital with a chief complaint of middle- to low-back pain with paresthesias of both lower limbs. She had a 2-year history of gait disturbance and lower-extremity bilateral numbness. Of note, she denied any history of trauma. On admission, the neurological examination showed muscle weakness of her iliopsoas and quadriceps femoris (4/5 strength), sensory disturbance, and hyperreflexia of the lower extremities. However, the bladder and bowel dysfunction were intact. Plain radiography revealed intervertebral disc space narrowing and Grade I anterior spondylolisthesis at T11/12. A magnetic resonance imaging scan revealed anterior spondylolisthesis and severe cord compression at T11 to T12, as well as high signal intensity in a T2-weighted image at T11/12. Axial T2-weighted MR imaging revealed right facet joint hyperintense signal suggesting joint effusion. And an axial CT showed bilateral facet joint osteoarthritis (Fig. 1). The patient underwent posterior transforaminal thoracic interbody fusion (TTIF).^[4]^ Under general anesthesia, the patient was placed on the operating table in the prone position, a standard midline posterior approach from T10 to T12 was used. After exposure, a gap was observed in the facet joint intraoperatively, indicating disruption of the joint capsule. A bilateral laminectomy of T11 up to the medial pedicle edge was performed and the entire inferior articular process and superior portion of the superior articular process on the left side were removed. The side of the spine selected for TTIF was based on preoperative symptoms and imaging. Access to the T11–12 disc space was achieved between the dura medially and the fatty tissue overlying the pleura laterally. The T11 nerve roots exited horizontally, lying cephalad to the disc space. Pituitary rongeurs, rasps, and curettes were used to remove disc material. Endplate removal and decortication provided an excellent graft bed adjacent to the anterior anulus. An 8-mm polyethyl-ether-ketone cage packed with local autograft was inserted into the disc space. After the contoured rods were set into the screw head, the locking plugs were tightened. The wound was irrigated, closed, sterile dressings applied and a hemovac drain was placed (Fig. 2). Postoperative images revealed that reduction of anterior displacement and resolution of the spinal cord compression (Fig. 3). Soon after the surgical procedures, the patient's back pain and lower-extremity numbness disappeared. She was subsequently discharged from the hospital. At 12-month follow-up, she was pain-free and had returned to full-time employment and activity without limitations. Solid instrumented fusion was achieved at 4-month follow-up. And MRI at 12-month follow-up revealed that an improvement in the high signal intensity on the T2-weighted image (Fig. 4). This study was approved by the Ethics Committee of Fuzhou Second Hospital affiliated to Xiamen University, and written informed consent was obtained from the patient.

## 3 Discussion and conclusions

The thoracic spine is stabilized in the anteroposterior direction by the rib cage and the facet joints, thus thoracic degenerative spondylolisthesis is more than uncommon. The exact mechanisms of degenerative thoracic spondylolisthesis is still not clear. In 2006, Shimada et al reported the first case of lower thoracic degenerative spondylithesis and reviewed other 3 similar cases reported in Japanese literature previously.^[1]^ The authors concluded that the increased pedicle-facet joint angle and disruption of the facet joint appeared to be the causes of the spondylolisthesis in the lower thoracic spine. Hsieh et al introduced 5 cases of lower thoracic degenerative spondylithesis with concomitant lumbar spondylosis, and believed that the diagnosis was challenging because the signs and symptoms were often subtle early in the course of the disease.^[2]^ He suggested that the facet joint laxity and disc degeneration were believed to be responsible for the development of thoracic spondylithesis. Takagi et al reported a case of thoracic spondylolisthesis and spinal cord compression in DISH.^[3]^ He believed that DISH resulted in the fusion of several spinal segments, and the unfused segments were more vulnerable to segmental instability and development of spondylolisthesis. Our patient is the first case report of thoracic degenerative spondylolisthesis in which the lower thoracic region was the only region involved. From the experience of the authors and review of the literature, disruption of joint capsule, instability with micromotion, and degenerative disc may contribute to this rare disease.

As the caliber of the thoracic spinal canal is relatively narrow and the spinal cord has a tenuous blood supply, severe neurological symptoms may develop and poor outcomes are likely if compression occurs and decompressive surgery is not performed promptly.^[5,6]^ Traditionally, thoracic myelopathy can be caused by ossification of the posterior longitudinal ligament, thoracic intervertebral disk herniation, posterior osteophytes, and ossification of the ligamentum flavum.^[5,6]^ As for thoracic degenerative spondylolisthesis, mild spondylolisthesis with relative canal stenosis and segmental instability with micromotion may gradually led to spinal cord compression.^[1–3]^

The surgical goals of thoracic degenerative spondylolisthesis were decompression of the spinal cord and stabilization of the thoracic spine.^[1–3]^ Of note, a good neurological recovery has an intimate relationship not only with adequate decompression but also with the stability of the spinal column.^[7]^ Reviewing the previous literature, the main treatment for thoracic degenerative spondylolisthesis has been surgical decompression using short-segment laminectomy in addition to pedicle screw fixation and posterolateral fusion.^[1–3]^ However, there are many shortcomings in this procedure, such as insufficient decompression, difficulties in restoring segmental stability, the height of intervertebral disc space and reduction.

In 2010 Machino et al introduced a new surgical approach called TTIF for thoracic spine lesions.^[4]^ TTIF involves posterior unilateral intervertebral joint excision as well as a foraminal zone approach, and dural sac and nerve roots can directly be visualized in 270° view. Thus, this technique is highly effective for decompression. In addition, a high level of safety is ensured because working space can be acquired in places without affecting neural tissues except nerve roots, and decompression can be performed without the need for retraction of the dural sac. It is possible to manipulate the interbody discs and reconstruct the anterior column through interbody bone transplantation. However, this surgical approach is not without shortcomings.^[7]^ Patients undergoing TTIF should be informed of the risk of pneumothorax. Additionally, the height and depth of the disc space is considerably smaller in the thoracic spine compared to the lumbar spine. Finally, retraction of the dura during the TTIF procedure is unsafe, and the possibility of iatrogenic spinal cord injury exists if any incidental contact with the dura occurs. Despite all the above shortcomings, TTIF enables posterior decompression, unilateral anterior decompression (total 270° decompression), and reconstruction of an anterior load support by interbody fusion. This procedure also enables early postoperative ambulation. TTIF can be a useful option for decompressive and reconstructive surgery of the mid and lower thoracic spine.^[7,8]^ In short, the TTIF approach can be a safe and useful method for providing interbody fusion and reconstructing the thoracic spine. As for thoracic degenerative spondylolisthesis, posterior decompression with posterolateral fusion or TTIF were the main treatment modalities, however, TTIF has its unique advantages because of sufficient decompression, immediate stability and high fusion rate.

In conclusion, thoracic degenerative spondylolisthesis is an extremely rare, and physicians should be alerted to the possibility of this rare entity. Disruption of joint capsule, instability with micromotion, and degenerative disc may contribute to this rare disease. Posterior decompression with posterolateral fusion or TTIF were the main treatment modalities, however, TTIF has its unique advantages because of sufficient decompression, immediate stability and high fusion rate.

## Author contributions

**Conceptualization:** Huafeng Wang, Fengfei Lin.

**Funding acquisition:** Huafeng Wang.

**Supervision:** Fengfei Lin.

**Validation:** Guiqing Liang, Boling Liu, Yuhan Lin.

**Writing – original draft:** Huafeng Wang.

**Writing – review & editing:** Huafeng Wang, Fengfei Lin, Guiqing Liang, Boling Liu, Yuhan Lin.

## References

[1] Shimada Y Kasukawa Y Miyakoshi N Hongo M Ando S Itoi E. Spondylolisthesis of the thoracic spine. Case report. J Neurosurg Spine 2006;4:415–8. 16703910 10.3171/spi.2006.4.5.415

[2] Hsieh PC Lee ST Chen JF. Lower thoracic degenerative spondylithesis with concomitant lumbar spondylosis. Clin Neurol Neurosurg 2014;118:21–5. 24529224 10.1016/j.clineuro.2013.11.019

[3] Takagi Y Yamada H Ebara H. Thoracic spondylolisthesis and spinal cord compression in diffuse idiopathic skeletal hyperostosis: a case report. J Med Case Rep 2017;11:90 doi: 10.1186/s13256-017-1252-0. 28363281 10.1186/s13256-017-1252-0 PMC5376279

[4] Machino M Yukawa Y Ito K. A new thoracic reconstruction technique “transforaminal thoracic interbody fusion”: a preliminary report of clinical outcomes. Spine (Phila Pa 1976) 2010;35:E1000–5. 20700083 10.1097/BRS.0b013e3181dc9153

[5] Hou X Sun C Liu X. Clinical features of thoracic spinal stenosis-associated myelopathy: a retrospective analysis of 427 cases. Clin Spine Surg 2016;29:86–9. 26885607 10.1097/BSD.0000000000000081

[6] Bouthors C Benzakour A Court C. Surgical treatment of thoracic disc herniation: an overview. Int Orthop 2019;43:807–16. 30406842 10.1007/s00264-018-4224-0

[7] Yu LJ Li WJ Guo SG. Transforaminal thoracic interbody fusion: treatment of thoracic myelopathy caused by anterior compression. Orthopade 2018;47:986–92. 29881916 10.1007/s00132-018-3588-6

[8] Liu FJ Chai Y Shen Y Xu JX Du W Zhang P. Posterior decompression with transforaminal interbody fusion for thoracic myelopathy due to ossification of the posterior longitudinal ligament and the ligamentum flavum at the same level. J Clin Neurosci 2013;20:570–5. 23313526 10.1016/j.jocn.2012.04.016

